# Prognostic Impact of Concomitant Beta-Blocker Use on Survival in EGFR-Mutant Metastatic Non-Small Cell Lung Cancer Patients Treated with Erlotinib

**DOI:** 10.3390/medicina61101843

**Published:** 2025-10-15

**Authors:** Oğuzhan Yıldız, Talat Aykut, Bahattin Engin Kaya, Ömer Genç, Ali Fuat Gürbüz, Fatih Saçkan, Melek Karakurt Eryılmaz, Mehmet Zahid Koçak, Murat Araz, Mehmet Artaç

**Affiliations:** 1 Department of Medical Oncology, Necmettin Erbakan University School of Medicine, Konya 42080, Turkey; talat_aykut@hotmail.com (T.A.); md.enginkaya@gmail.com (B.E.K.); omergenc58@hotmail.com (Ö.G.); dr.alifuatgurbuz@hotmail.com (A.F.G.); drangelkarakurt@hotmail.com (M.K.E.); mehmetzahidkocak@hotmail.com (M.Z.K.); zaratarum@yahoo.com (M.A.); mehmetartac@yahoo.com (M.A.); 2 Denizli State Hospital, Denizli 20010, Turkey; drfatihsackan@gmail.com

**Keywords:** beta-blocker, erlotinib, EGFR-mutant metastatic non-small cell lung cancer, survival

## Abstract

*Background and Objectives*: Erlotinib, a tyrosine kinase inhibitor (TKI), is an established therapy for patients with metastatic non-small cell lung cancer (NSCLC) harboring epidermal growth factor receptor (EGFR) mutations. Preclinical and clinical evidence suggests that chronic stress, mediated through β-adrenergic signaling, promotes tumor progression, angiogenesis, and therapy resistance. Furthermore, interactions between β-adrenergic signaling and EGFR pathways have been hypothesized to negatively influence treatment responses. Based on this rationale, we investigated whether concomitant beta-blocker use may improve survival outcomes in EGFR-mutant NSCLC patients treated with erlotinib. *Materials and Methods*: This retrospective analysis included 103 patients with metastatic EGFR-mutant NSCLC who received erlotinib. Patients were classified according to concurrent beta-blocker use, defined as continuous therapy for at least six months prior to erlotinib initiation, prescribed for cardiovascular indications. Progression-free survival (PFS) and overall survival (OS) were compared between beta-blocker users and non-users. *Results*: Patients receiving erlotinib with concomitant beta-blocker therapy achieved a median PFS (mPFS) of 21.4 months (95% CI, 13.1–29.7), compared with 9.7 months (95% CI, 6.7–12.7) in non-users (*p* = 0.003). Median OS (mOS) was 32.4 months (95% CI, 14.8–50.0) in the beta-blocker group versus 19.9 months (95% CI, 14.8–25.0) in the non-beta-blocker group (*p* = 0.010). Multivariate Cox regression confirmed beta-blocker use as an independent prognostic factor for both PFS (*p* = 0.004) and OS (*p* = 0.014). *Conclusions*: Concomitant beta-blocker use was associated with significantly prolonged survival in patients with EGFR-mutant metastatic NSCLC receiving erlotinib. These findings support the hypothesis that β-adrenergic inhibition enhances the efficacy of EGFR-targeted therapy. Prospective studies are warranted to validate these results and to further elucidate the underlying biological mechanisms.

## 1. Introduction

NSCLC is the leading cause of cancer-related mortality worldwide. It has been determined that this accounts for approximately 85% of all lung cancer cases. The frequency of EGFR mutations in NSCLC varies according to ethnicity, smoking status, and tumor histology, ranging from 10 to 15% in Western populations to up to 50% in certain Asian cohorts [1,2]. Erlotinib, a first-generation EGFR TKI, has demonstrated significant clinical benefit in patients with EGFR-mutant NSCLC and has been shown to improve PFS compared to standard chemotherapy [3]. Nevertheless, despite the initial therapeutic efficacy, the development of acquired resistance to EGFR-TKIs remains a major clinical problem. Recent studies have indicated that tumor progression and the development of resistance to anti-EGFR therapies may be associated with neuroendocrine and stress-related pathways. It is well established that chronic psychological stress activates the sympathetic nervous system, leading to the release of norepinephrine and epinephrine. These mediators stimulate processes integral to tumor cell proliferation, migration, invasion, and angiogenesis through β-adrenergic receptor signaling [4]. Preclinical data have suggested a potential interaction between EGFR and β-adrenergic signaling pathways, which has been shown to contribute to the development of resistance mechanisms, particularly in EGFR-mutant tumors [5]. Beta-blockers are widely used in the treatment of cardiovascular diseases, including conditions such as hypertension and ischemic heart disease [6]. These agents block the effects of sympathetic stimulation on β-adrenergic receptors. Current evidence suggests that activation of the β-adrenergic pathway may adversely affect EGFR-targeted therapies by promoting intracellular proliferative and anti-apoptotic signaling, ultimately leading to therapeutic resistance [7]. In our study, patients were not directly treated with beta-blockers; however, significantly better clinical outcomes were observed in EGFR-mutant NSCLC patients who were already receiving beta-blockers for cardiovascular indications. This striking finding provides a strong biological and clinical signal that beta-blocker use in patients receiving EGFR-TKI therapy, in the absence of contraindications, may substantially improve survival outcomes. Nevertheless, data regarding the impact of beta-blocker use on survival outcomes in EGFR-mutant NSCLC patients receiving TKI therapy are limited. If these results are confirmed by prospective and randomized studies, the addition of beta-blockers to EGFR-TKI therapy may provide an important innovation in standard treatment algorithms and contribute to achieving longer survival in this patient population.

## 2. Materials and Methods

This study is a retrospective cohort analysis. EGFR-mutated NSCLC patients receiving erlotinib treatment were included in the study. Medical records of eligible patients treated between 10 December 2010, and 1 January 2025, were retrieved from institutional archives and reviewed. Inclusion criteria required a confirmed diagnosis of EGFR mutation, receipt of erlotinib therapy, and the availability of complete and regularly maintained clinical records in the hospital database. In addition, patients with regular medication reports and treatment prescriptions recorded in the national electronic prescription system, and who had been receiving beta-blocker therapy continuously for at least six months, were included in the beta-blocker group. This approach ensured the objective verification of treatment continuity and increased the reproducibility of retrospective data. Patients were also evaluated for a history of hypertension and ischemic heart disease. Clinical evidence indicates that sustained β-adrenergic inhibition over several months is required to achieve meaningful biological modulation, as observed in chronic cardiovascular conditions such as heart failure. For this reason, a six-month threshold was chosen. This criterion was applied to ensure that beta-blocker use was prescribed for cardiovascular indications independent of the oncologic treatment process, thereby minimizing indication bias. Baseline demographic, clinical, and treatment-related parameters were compared between patients receiving concomitant beta-blocker therapy and those who did not. These parameters included age, sex, smoking history, Eastern Cooperative Oncology Group (ECOG) performance status at diagnosis, comorbidities, stage at diagnosis (metastatic vs. non-metastatic), metastatic site distribution (bone, liver, brain, adrenal), receipt of primary tumor surgery, radiologic response to erlotinib, and administration of post-TKI systemic therapy.

### Statistical Analysis

IBM SPSS Statistics version 20 was used for statistical analyses. Descriptive statistics were expressed as counts, percentages, and proportions. Categorical variables were compared using the chi-square test. The distribution of continuous variables was assessed with the Kolmogorov–Smirnov test. The independent samples *t*-test was used for normally distributed variables, and the Mann–Whitney U test was applied for non-normally distributed variables. PFS and OS were calculated using the Kaplan–Meier method, and differences between groups were compared with the log-rank test. A two-sided *p* < 0.05 was considered statistically significant. To evaluate the impact of clinical variables, univariate Cox regression analyses were first performed. Clinically relevant variables (such as stage at diagnosis and primary tumor surgery) and variables with *p* < 0.05 in the univariate analysis were included in the multivariate Cox regression model. A forward stepwise selection method was used, which aimed to reduce the risk of overfitting due to the limited sample size while controlling for the effect of significant confounders. A post hoc power analysis was conducted to assess whether the sample size was sufficient to detect the observed survival differences. The hazard ratio (HR) for OS was 0.548, and the HR for PFS was 0.508. At a significance level of 5%, the statistical power was calculated as 0.81 for both endpoints. These results demonstrated that the study had sufficient power to show clinically meaningful survival differences and that the probability of a type II error was low.

## 3. Results

A total of 103 patients with EGFR-mutant metastatic NSCLC who received erlotinib treatment were included in the study. Of these, 35 patients (34.0%) received concomitant beta-blocker therapy, while 68 patients (66.0%) did not. The groups were compared with respect to demographic and clinical characteristics. Among beta-blocker users, 19 patients (54.3%) were ≥65 years, compared with 30 patients (44.1%) in the non–beta-blocker group (*p* = 0.328). The proportion of female patients was 20 (57.1%) in the beta-blocker group and 39 (57.4%) in the non–beta-blocker group (*p* = 0.984). The proportion of never-smokers was 24 (68.6%) in the beta-blocker group and 46 (67.6%) in the non–beta-blocker group (*p* = 0.924). ECOG performance status 0 at diagnosis was observed in 15 patients (42.9%) receiving beta-blockers and in 25 patients (36.8%) not receiving beta-blockers (*p* = 0.548). The distribution of first-line treatment was similar: in the beta-blocker group, 17 patients (48.6%) received chemotherapy and 18 patients (51.4%) received erlotinib; in the non–beta-blocker group, 35 patients (51.5%) received chemotherapy and 33 patients (48.5%) received erlotinib (*p* = 0.780). Following TKI progression, subsequent systemic therapy was administered to 7 patients (20.0%) in the beta-blocker group and 10 patients (14.7%) in the non–beta-blocker group (*p* = 0.493). Comorbid conditions were present in 22 patients (62.9%) receiving beta-blockers and 23 patients (33.8%) not receiving beta-blockers (*p* = 0.005). At diagnosis, non-metastatic disease was observed in 13 patients (37.1%) in the beta-blocker group and in 5 patients (7.4%) in the non–beta-blocker group (*p* < 0.001). Primary tumor surgery was performed in 10 patients (28.6%) in the beta-blocker group and 4 patients (5.9%) in the non–beta-blocker group (*p* = 0.004). With respect to metastatic sites, liver metastasis was identified in 1 patient (2.9%) receiving beta-blockers and 16 patients (23.5%) not receiving beta-blockers (*p* = 0.007). Bone metastasis was present in 17 patients (48.6%) in the beta-blocker group and 46 patients (67.6%) in the non–beta-blocker group (*p* = 0.060). Adrenal metastasis was found in 5 patients (14.3%) and 17 patients (25.0%), respectively (*p* = 0.209), and brain metastasis in 5 patients (14.3%) and 12 patients (17.6%), respectively (*p* = 0.663). Regarding treatment response, radiologic response to erlotinib was observed in 24 patients (68.6%) in the beta-blocker group and 34 patients (50.0%) in the non–beta-blocker group (*p* = 0.072) (Table 1).

Among patients receiving erlotinib with concomitant beta-blockers, the median OS was 31.3 months (95% CI, 8.3–54.3; n = 8) for nebivolol, 67.5 months (95% CI, 0.0–139.9; n = 11) for metoprolol, and 23.5 months (95% CI, 14.2–32.7; n = 17) for bisoprolol. There was no statistically significant difference among the groups (log-rank *p* = 0.471). mPFS was 21.4 months (95% CI, 13.1–29.7) in patients receiving beta-blockers and 9.7 months (95% CI, 6.7–12.7) in those who did not (*p* = 0.003). By age group, median mPFS was 9.4 months for patients < 65 years and 17.0 months for those ≥ 65 years (*p* = 0.150). Median mPFS was 15.4 months in females and 11.3 months in males (*p* = 0.531). Among patients without comorbidities, median mPFS was 12.4 months, compared with 12.8 months in those with comorbidities (*p* = 0.367). Never-smokers had a median mPFS of 15.4 months, whereas smokers had 9.2 months (*p* = 0.150). Median mPFS was 17.2 months in patients with ECOG 0 performance status and 12.4 months in those with ECOG ≥ 1 (*p* = 0.106). Patients diagnosed with non-metastatic disease had a median mPFS of 22.5 months, compared with 11.4 months in those with metastatic disease (*p* = 0.034). Median mPFS was 12.2 months in patients without primary tumor surgery and 22.5 months in those who underwent surgery (*p* = 0.070). With respect to metastatic sites, median mPFS was 24.4 months in patients without bone metastases and 9.8 months in those with bone metastases (*p* < 0.001). Patients without liver metastases had a median mPFS of 14.3 months, compared with 6.9 months in those with liver metastases (*p* = 0.136). Median mPFS was 12.4 months in patients without adrenal metastases and 12.5 months in those with adrenal metastases (*p* = 0.774). Median mPFS was 12.5 months in patients without brain metastases and 10.1 months in those with brain metastases (*p* = 0.398). According to first-line treatment, median mPFS was 11.3 months in patients receiving chemotherapy and 16.3 months in those receiving erlotinib (*p* = 0.590). Median mPFS was 4.2 months in patients without a radiologic response to TKI and 22.5 months in those with a response (*p* < 0.001). Patients who did not receive post-TKI systemic therapy had a median mPFS of 11.4 months, whereas those who received post-TKI therapy had 24.4 months (Table 2).

In the survival analysis, mOS was 19.9 months (95% CI, 14.8–25.0) in patients not receiving beta-blockers and 32.4 months (95% CI, 14.8–50.0) in those receiving beta-blockers (*p* = 0.010). By age group, median mOS was 23.5 months for patients < 65 years and 23.0 months for those ≥ 65 years (*p* = 0.723). Median mOS was 34.7 months in females and 18.9 months in males (*p* = 0.209). Among patients without comorbidities, median mOS was 30.2 months, compared with 20.7 months in those with comorbidities (*p* = 0.464). Never-smokers had a median mOS of 30.2 months, whereas smokers had 18.2 months (*p* = 0.027). Median mOS was 39.2 months in patients with ECOG 0 performance status and 20.5 months in those with ECOG ≥ 1 (*p* = 0.022). Patients diagnosed with non-metastatic disease had a median mOS of 30.2 months, compared with 23.0 months in those with metastatic disease (*p* = 0.035). Median mOS was 22.3 months in patients without primary tumor surgery and 31.8 months in those who underwent surgery (*p* = 0.033). With respect to metastatic sites, median mOS was 52.5 months in patients without bone metastases and 18.2 months in those with bone metastases (*p* < 0.001). Patients without liver metastases had a median mOS of 27.8 months, compared with 8.4 months in those with liver metastases (*p* = 0.016). Median mOS was 23.0 months in patients without adrenal metastases and 23.1 months in those with adrenal metastases (*p* = 0.789). Median mOS was 26.3 months in patients without brain metastases and 9.7 months in those with brain metastases (*p* = 0.012). According to first-line treatment, median mOS was 20.5 months in patients receiving chemotherapy and 27.9 months in those receiving erlotinib (*p* = 0.264). Median mOS was 13.2 months in patients without a radiologic response to TKI and 41.6 months in those with a response (*p* < 0.001). Patients who did not receive post-TKI systemic therapy had a median mOS of 20.7 months, whereas those who received post-TKI therapy had 48.7 months (Table 2).

In the univariate Cox regression analysis for mPFS, smoking history was not significantly associated with outcome (HR, 0.722; 95% CI, 0.463–1.127; *p* = 0.152). ECOG performance status > 0 at diagnosis did not show a significant effect (HR, 0.701; 95% CI, 0.455–1.081; *p* = 0.108). Primary tumor surgery was not significantly associated with mPFS (HR, 1.759; 95% CI, 0.947–3.268; *p* = 0.074). Concomitant beta-blocker use was significantly associated with improved mPFS (HR, 0.508; 95% CI, 0.321–0.803; *p* = 0.004). The presence of metastatic disease at diagnosis was associated with shorter mPFS (HR, 0.545; 95% CI, 0.309–0.962; *p* = 0.036). Variables with a *p*-value < 0.05 in the univariate analysis were included in the multivariate Cox regression analysis. Using a forward stepwise selection method (χ^2^(1) = 8.679; *p* = 0.003), beta-blocker use remained the only independent predictor of improved mPFS in the final model (HR, 0.508; 95% CI, 0.321–0.803; *p* = 0.004) (Table 3).

In the univariate Cox regression analysis for OS, smoking history was significantly associated with worse OS (HR, 1.685; 95% CI, 1.057–2.686; *p* = 0.028). An ECOG performance status > 0 at diagnosis was also associated with poorer outcomes (HR, 1.681; 95% CI, 1.072–2.637; *p* = 0.024). Patients who underwent primary tumor surgery had improved OS (HR, 0.459; 95% CI, 0.220–0.956; *p* = 0.037). Concomitant beta-blocker use was significantly associated with prolonged OS (HR, 0.537; 95% CI, 0.333–0.866; *p* = 0.011). The presence of metastatic disease at diagnosis was linked to shorter OS (HR, 1.966; 95% CI, 1.038–3.722; *p* = 0.038). Variables with a *p*-value < 0.05 in the univariate analysis were included in the multivariate Cox regression analysis. Using a forward stepwise method (χ^2^(2) = 11.492; *p* = 0.003), two independent predictors of OS were identified: ECOG performance status > 0 at diagnosis (HR, 1.644; 95% CI, 1.048–2.579; *p* = 0.030) and beta-blocker use (HR, 0.548; 95% CI, 0.340–0.883; *p* = 0.014). These findings indicate that beta-blocker use was independently associated with improved OS in patients with EGFR-mutant NSCLC treated with erlotinib (Table 4). The concordance index (C-index) of the final multivariable Cox model was 0.662, indicating moderate discriminative ability.

**Figure 1:**
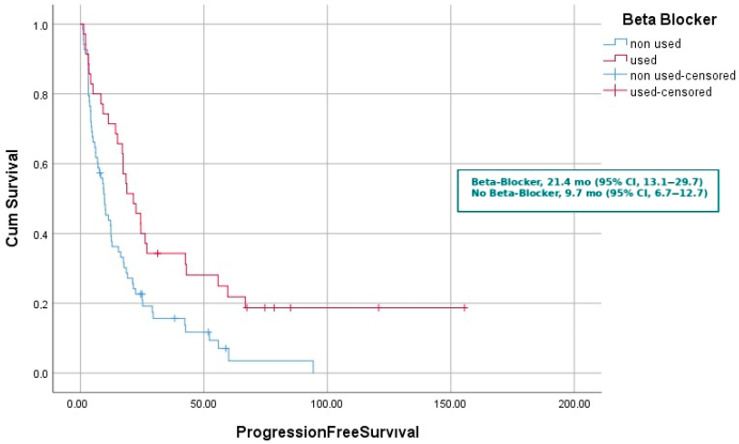
Kaplan–Meier curves Progression Free Survival according to concomitant beta-blocker use and non-use.

The survival analysis revealed a statistically significant difference in PFS and OS according to beta-blocker use. Patients receiving concomitant beta-blocker therapy had a median PFS of 21.4 months (95% CI, 13.1–29.7), compared with 9.7 months (95% CI, 6.7–12.7) in those not receiving beta-blockers (*p* = 0.003) (Figure 1).

**Figure 2:**
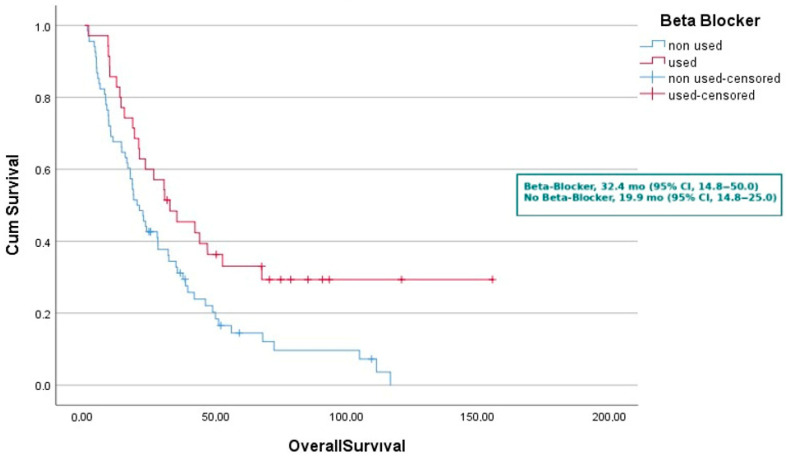
Kaplan–Meier curves Overall Survival according to concomitant beta-blocker use and non-use.

Similarly, the median OS was 32.4 months (95% CI, 14.8–50.0) in the beta-blocker group, significantly longer than 19.9 months (95% CI, 14.8–25.0) in the non-beta-blocker group (*p* = 0.010) (Figure 2). These findings were further supported by the Kaplan–Meier survival curves, which demonstrated a marked separation in survival probabilities over time, favoring patients who received beta-blockers. To further validate the strength of the observed survival differences, a post hoc power analysis was performed. This analysis confirmed that the study had a statistical power of 0.81 for both PFS and OS comparisons, indicating a low likelihood of type II error and reinforcing the reliability of the findings. The observed differences suggest a potential clinical benefit of beta-adrenergic blockade in EGFR-mutant NSCLC patients treated with erlotinib.

## 4. Discussion

The present study demonstrated that concomitant use of beta-blockers with erlotinib therapy in patients with metastatic EGFR-mutant NSCLC was associated with significantly prolonged mPFS and mOS, and that this effect was retained as an independent prognostic factor in multivariate analysis. These findings support the critical role of the beta-adrenergic signaling pathway in regulating biological processes that drive tumor progression. Prior studies have reported that the adrenergic system actively contributes to fundamental aspects of cancer biology, including angiogenesis, cellular proliferation, invasion, and therapeutic resistance. In particular, stimulation of beta-adrenergic receptors has been shown to increase intracellular cAMP levels, leading to activation of downstream pathways via protein kinase A, which intersect with EGFR-mediated MAPK and PI3K–AKT signaling, thereby facilitating tumor cell proliferation, metastatic potential, and the development of resistance to anti-EGFR therapies. In EGFR-mutant NSCLC, resistance to TKIs encompasses a spectrum of multilayered biological processes, including the emergence of secondary mutations within the receptor tyrosine kinase domain, activation of alternative receptor tyrosine kinases through amplification or rearrangement, reprogramming of intracellular signaling networks, and phenotypic histologic transformation of tumor cells. These complex resistance interactions ultimately lead to the loss of therapeutic response over time and exert a substantial impact on clinical outcomes. While next-generation targeted agents address some of these resistance mechanisms, their high costs and protracted development timelines limit widespread clinical implementation. In this context, the strategy of drug repurposing re-evaluating well-established agents with favorable safety profiles offers the potential to enhance therapeutic benefit without increasing treatment cost.

It has been demonstrated that beta-blockers have the capacity to influence responses to oncologic therapies by interacting with multiple therapeutic pathways through diverse mechanisms. In a study conducted by Korkmaz et al., concomitant use of beta-blockers with VEGF-targeted therapies in patients with metastatic renal cell carcinoma was found to be associated with significant improvements in both OS and PFS [8]. Similarly, Koçak et al. reported that in patients with metastatic colorectal cancer, the addition of beta-blockers to bevacizumab-based chemotherapy prolonged PFS and OS, irrespective of the type of beta-blocker used [9]. Aydıner et al. also demonstrated an association between beta-blocker use in combination with chemotherapy and OS in patients with metastatic NSCLC; however, this benefit was not sustained in multivariate analysis [10]. In the meta-analysis by Ichiro Tsuboi and colleagues, the use of beta blockers in combination with TKI was shown to significantly improve OS all survival in patients with metastatic renal cell carcinoma [11]. In a study conducted by Fiala and colleagues, the use of beta blockers in combination with immunotherapy and TKIs was shown to have no effect on OS in patients with metastatic renal cell carcinoma [12]. These differing results obtained in different cancers suggest that the effects of beta blockers may also reflect their different effects on resistance mechanisms, beyond their interaction with direct TKIs. Indeed, studies conducted to date have demonstrated that the adrenergic system plays a role in the development of anti-EGFR treatment resistance.

A substantial body of research has evaluated the potential benefits of concomitant beta-blocker use in various cancer types. In the context of malignant melanoma, prostate cancer, and breast cancer, the observed efficacy has been attributed to the inhibition of angiogenesis and metastasis. In contrast, our study suggests that in patients with EGFR-mutant NSCLC receiving erlotinib, beta-blockers may confer additional benefits by modulating intracellular signaling pathways implicated in therapeutic resistance, such as MAPK and PI3K–AKT, downstream of EGFR activation [13,14,15]. According to recent research, resistance to anti-EGFR therapies is also influenced by the beta-adrenergic system. As demonstrated by Chang et al., a prior history of beta-blocker use was associated with prolonged median PFS and median OS in patients with lung adenocarcinoma receiving first-line EGFR-TKI therapy [4]. This finding supports the hypothesis that beta-adrenergic signaling may contribute to the emergence of resistance mechanisms against anti-EGFR therapies. Adrenergic blockade has the potential to enhance the efficacy of anti-EGFR therapy by inhibiting resistance mechanisms. In this context, beta-blockers may play a critical role in restoring treatment sensitivity, slowing tumor growth, and suppressing angiogenesis. Given that beta-blockers are widely used in routine clinical practice and have well-established safety profiles, their ability to provide meaningful therapeutic benefits when combined with anti-EGFR therapy represent a particularly attractive opportunity. This biologically plausible hypothesis underscores the potential of beta-blockers to improve outcomes without adding substantial treatment-related toxicities. Prospective, controlled clinical trials are warranted to confirm this concept and to clarify whether such an approach could ultimately influence therapeutic strategies, particularly in high-risk or treatment-resistant patient populations.

Several limitations of this study should be acknowledged. First, it was designed as a retrospective analysis with a relatively small sample size, particularly in the beta-blocker group. Nevertheless, the presence of statistically significant findings, supported by post hoc power analysis, strengthens the reliability of the results. In addition, there were baseline imbalances between patients who did and did not receive beta-blockers, most notably with respect to disease stage at diagnosis and the rates of primary tumor resection. These differences may have influenced survival outcomes independently of beta-blocker use. Although multivariate adjustments were performed, the possibility of residual confounding cannot be entirely excluded. Furthermore, the single-center nature of the study may limit the generalizability of the findings, and the relatively small sample size increases the risk of sampling bias. Even so, the clinical relevance of these observations remains noteworthy, and confirmation in larger, prospective, multicenter studies would be appropriate.

Standartised treatment protocols, prospective multicenter studies, and larger, well-balanced cohorts will be needed to improve the external validity of these observations. These results offer a strong biological and clinical justification for additional research in the context of EGFR-mutant NSCLC, given the well-established safety profile, widespread clinical availability, and affordable price of beta-blockers. Such an approach not only presents a readily accessible opportunity to achieve significant clinical benefit without imposing additional toxicity, but it also has the potential to inform the development of novel therapeutic strategies. To confirm these findings and clarify the underlying biological mechanisms, prospective clinical trials in the future will be crucial.

## 5. Conclusions

Our findings indicate that patients with EGFR-mutant metastatic NSCLC receiving erlotinib derive significantly improved PFS and OS when concomitant β-adrenergic blockade is present. These observations suggest that modulation of the β-adrenergic signaling pathway may augment the efficacy of EGFR-targeted therapy and potentially delay the emergence of resistance mechanisms. Given the widespread clinical use, favorable safety profile, and low cost of β-blockers, incorporation of β-adrenergic inhibition into treatment paradigms represents a feasible and readily implementable strategy. Nevertheless, prospective randomized trials are needed to validate these findings and to more precisely define the patient subgroups most likely to benefit from this approach.

## Figures and Tables

**Table 1:** Baseline Clinical Characteristics According to beta-blocker Use Among EGFR-Mutant NSCLC Patients Receiving Erlotinib.

Variable	Total (n = 103)	No Beta-Blocker (n = 68)	Beta-Blocker (n = 35)	*p*-Value
**Age Group**				**0.328**
<65	54 (52.4%)	38 (55.9%)	16 (45.7%)	
≥65	49 (47.6%)	30 (44.1%)	19 (54.3%)	
**Sex**				**0.984**
Female	59 (57.3%)	39 (57.4%)	20 (57.1%)	
Male	44 (42.7%)	29 (42.6%)	15 (42.9%)	
**Comorbid Conditions**				**0.005**
Absent	58 (56.3%)	45 (66.2%)	13 (37.1%)	
Present	45 (43.7%)	23 (33.8%)	22 (62.9%)	
**Smoking History**				**0.924**
Never-smoker	70 (68.0%)	46 (67.6%)	24 (68.6%)	
Smoker	33 (32.0%)	22 (32.4%)	11 (31.4%)	
**ECOG at Diagnosis**				**0.548**
ECOG 0	40 (38.8%)	25 (36.8%)	15 (42.9%)	
ECOG ≥ 1	63 (61.2%)	43 (63.2%)	20 (57.1%)	
**Metastasis at Diagnosis**				**<0.001**
Non-metastatic	18 (17.5%)	5 (7.4%)	13 (37.1%)	
Metastatic	85 (82.5%)	63 (92.6%)	22 (62.9%)	
**Primary Tumor Surgery**				**0.004**
No	89 (86.4%)	64 (94.1%)	25 (71.4%)	
Yes	14 (13.6%)	4 (5.9%)	10 (28.6%)	
**Bone Metastasis**				**0.060**
Absent	40 (38.8%)	22 (32.4%)	18 (51.4%)	
Present	63 (61.2%)	46 (67.6%)	17 (48.6%)	
**Liver Metastasis**				**0.007**
Absent	86 (83.5%)	52 (76.5%)	34 (97.1%)	
Present	17 (16.5%)	16 (23.5%)	1 (2.9%)	
**Adrenal Metastasis**				**0.209**
Absent	81 (78.6%)	51 (75.0%)	30 (85.7%)	
Present	22 (21.4%)	17 (25.0%)	5 (14.3%)	
**Brain Metastasis**				**0.663**
Absent	86 (83.5%)	56 (82.4%)	30 (85.7%)	
Present	17 (16.5%)	12 (17.6%)	5 (14.3%)	
**1st-Line Treatment**				**0.780**
Chemotherapy	52 (50.5%)	35 (51.5%)	17 (48.6%)	
Erlotinib	51 (49.5%)	33 (48.5%)	18 (51.4%)	
**Response to TKI**				**0.072**
Non-responsive	45 (43.7%)	34 (50.0%)	11 (31.4%)	
Responsive	58 (56.3%)	34 (50.0%)	24 (68.6%)	
**Post-TKI Treatment**				**0.493**
No	86 (83.5%)	58 (85.3%)	28 (80.0%)	
Yes	17 (16.5%)	10 (14.7%)	7 (20.0%)	

**Table 2:** FS and OS Based on Clinical and Treatment Characteristics in EGFR-Mutant mNSCLC.

Variable	Median PFS (Months)	95% Confidence Interval	*p*-Value	Variable	Median OS (Months)	95% Confidence Interval	*p*-Value
**Beta-Blocker Use**			**0.003**	**Beta-Blocker Use**			**0.010**
Not used	9.7	6.7–12.7		Not used	19.9	14.8–25.0	
Used	21.4	13.1–29.7		Used	32.4	14.8–50.0	
Age Group			**0.150**	Age Group			**0.723**
<65 years	9.4	5.5–13.3		<65 years	23.5	12.2–34.8	
≥65 years	17	11.6–22.3		≥65 years	23.0	14.4–31.6	
Sex			**0.531**	Sex			**0.209**
Female	15.4	10.2–20.6		Female	34.7	20.5–48.9	
Male	11.3	4.7–18		Male	18.9	13.3–24.5	
Comorbid Conditions			**0.367**	Comorbid Conditions			**0.464**
Absent	12.4	6.8–18		Absent	30.2	16.9–43.5	
Present	12.8	36–22		Present	20.7	16.0–25.4	
Smoking History			**0.150**	Smoking History			**0.027**
Never-smoker	15.4	10.7–20.1		Never-smoker	30.2	14.3–46.1	
Smoker	9.2	1.7–16.6		Smoker	18.2	8.6–27.8	
ECOG at Diagnosis			**0.106**	ECOG at Diagnosis			**0.022**
ECOG 0	17.2	6.6–27.8		ECOG 0	39.2	23.6–54.9	
ECOG ≥ 1	12.4	9–15.7		ECOG ≥ 1	20.5	15.1–25.9	
Metastasis at Diagnosis			**0.034**	Metastasis at Diagnosis			**0.035**
Non-metastatic	22.5	0–71.9		Non-metastatic	30.2	0–75	
Metastatic	11.4	8.6–14.1		Metastatic	23.0	14.1–31.9	
Primary Tumor Surgery			**0.070**	Primary Tumor Surgery			**0.033**
No	12.2	7.8–16.7		No	22.3	14.8–29.8	
Yes	22.5	0–68.9		Yes	31.8	7.1–56.4	
Bone Metastasis			**<0.001**	Bone Metastasis			**<0.001**
Absent	24.4	15.4–33.4		Absent	52.5	17.5–87.4	
Present	9.8	7.4–12.2		Present	18.2	14.2–22.2	
Liver Metastasis			**0.136**	Liver Metastasis			**0.016**
Absent	14.3	9.7–18.8		Absent	27.8	18.6–37.0	
Present	6.9	0–14.1		Present	8.4	0–16.8	
Adrenal Metastasis			**0.774**	Adrenal Metastasis			**0.789**
Absent	12.4	7.1–17.6		Absent	23.0	13.5–32.6	
Present	12.5	0–26.2		Present	23.1	8.3–37.9	
Brain Metastasis			**0.398**	Brain Metastasis			**0.012**
Absent	12.5	8–17		Absent	26.3	19.0–33.5	
Present	10.1	0–24.2		Present	9.7	0–23.7	
1st-Line Treatment			**0.590**	1st-Line Treatment			**0.264**
Chemotherapy	11.3	6.6–16.1		Chemotherapy	20.5	14.6–26.4	
Erlotinib	16.3	9.8–22.8		Erlotinib	27.9	15.2–40.6	
Response to TKI			**<0.001**	Response to TKI			**<0.001**
Non-responsive	4.2	3.6–4.8		Non-responsive	13.2	4.6–21.8	
Responsive	22.5	16.1–28.9		Responsive	41.6	32.0–51.2	
Post-TKI Treatment			**0.031**	Post-TKI Treatment			**0.031**
Not received	11.4	6.3–16.4		Not received	20.7	15.7–25.8	
Received	24.4	7.3–41.4		Received	48.7	18.9–78.4	
Median Progression Free Survival				Median Overall Survival			

**Table 3:** Univariate and Multivariate Predictors of Progression Free Survival in EGFR-Mutant NSCLC.

	Univariate		Multivariate	
Variable	HR (95% CI)	*p*-Value	HR (95% CI)	*p*-Value
Smoking History	0.722 (0.463–1.127)	0.152		
ECOG > 0 at Diagnosis	0.701 (0.455–1.081)	0.108		
Primary Tumor Surgery	1.759 (0.947–3.268)	0.074		
Beta-Blocker Use	0.508 (0.321–0.803)	0.004	0.508 (0.321–0.803)	0.004
Metastasis Status	0.545 (0.309–0.962)	0.036		

Parameters found to be significantly associated with PFS in the univariate analysis were included in the multivariate analysis. The Cox regression analysis was performed using the forward stepwise method (χ^2^(1) = 8.679, *p* = 0.003), and the final model (step 1) is presented in the table. Abbreviations: HR = Hazard Ratio, CI = Confidence Interval.

**Table 4:** Univariate and Multivariate Predictors of Overall Survival in EGFR-Mutant NSCLC.

	Univariate		Multivariate	
Variable	HR (95% CI)	*p*-Value	HR (95% CI)	*p*-Value
Smoking History	1.685 (1.057–2.686)	0.028		
ECOG > 0 at Diagnosis	1.681 (1.072–2.637)	0.024	1.644 (1.048–2.579)	0.030
Primary Tumor Surgery	0.459 (0.220–0.956)	0.037		
Beta-Blocker Use	0.537 (0.333–0.866)	0.011	0.548 (0.340–0.883)	0.014
Metastasis at diagnosis	1.966 (1.038–3.722)	0.038		
ECOG > 0 at Diagnosis	1.644 (1.048–2.579)	0.030		
Beta-Blocker Use	0.548 (0.340–0.883)	0.014		

Variables with a *p*-value < 0.05 in the univariate analysis were included in the multivariate Cox regression analysis. A forward stepwise method was used (χ^2^(2) = 11.492, *p* = 0.003), and the final model (step 2) is shown in the table. Abbreviations: HR = Hazard Ratio; CI = Confidence Interval.

## Data Availability

The data that support the findings of this study are available on request from the corresponding author. The data are not publicly available due to privacy or ethical restrictions.

## Abbreviations

The following abbreviations are used in this manuscript:

TKI	Tyrosine kinase inhibitor
mNSCLC	metastatic non-small cell lung cancer
PFS	Progression-free survival
OS	Overall Survival
